# Effects of Adapted Physical Activity on White Matter Integrity in Patients with Schizophrenia

**DOI:** 10.3390/brainsci14070710

**Published:** 2024-07-15

**Authors:** Elise Leroux, Laura Masson, Maxime Tréhout, Sonia Dollfus

**Affiliations:** 1 “Physiopathology and Imaging of Neurological Disorders” PhIND, UMR-S U1237, INSERM, GIP Cyceron, 14000 Caen, France; masson@cyceron.fr (L.M.); trehout-m@chu-caen.fr (M.T.); dollfus@cyceron.fr (S.D.); 2 CHU de Caen Normandie, Centre Esquirol, Service de Psychiatrie Adulte, 14000 Caen, France; 3 Normandie Univ, Université de Caen Normandie, UFR de Santé, 14000 Caen, France; 4 Fédération Hospitalo-Universitaire “Améliorer le Pronostic des Troubles Addictifs et Mentaux par une Médecine Personnalisée (A2M2P)“, 14000 Caen, France

**Keywords:** schizophrenia, adapted physical activity, TBSS, white matter, MRI, negative symptoms

## Abstract

Schizophrenia is associated with changes in white matter (WM) integrity and with reduced life expectancy, in part because of the cardiometabolic side effects of antipsychotics. Physical activity (PA) has emerged as a candidate lifestyle intervention that is safe and effective. The study aimed to assess how an adapted PA program delivered remotely by web (e-APA) improved WM integrity in patients with schizophrenia (SZPs) and healthy controls (HCs) and to evaluate associations among WM integrity, cardiorespiratory fitness, and symptom severity. This longitudinal study was conducted over 16 weeks with 31 participants (18 SZPs and 13 HCs). Diffusion tensor imaging and tract-based spatial statistics were employed to assess WM integrity. Cardiorespiratory fitness was measured by maximal oxygen uptake (VO_2_max), and assessments for clinical symptoms included the Positive and Negative Syndrome Scale, Self-evaluation of Negative Symptoms and the Brief Negative Syndrome Scale (BNSS). Only the SZPs had significantly increased WM integrity after the e-APA program, with increased fractional anisotropy and decreased radial diffusivity in fasciculi involved in motor functions and language process. Furthermore, decreased negative symptoms assessed with BNSS were associated with greater WM integrity following the program. These findings suggest that e-APA may improve WM integrity abnormalities and support e-APA as a promising therapeutic strategy.

## 1. Introduction

Schizophrenia is associated with changes in white matter (WM) structure and integrity within a diffuse cortico-subcortical network mainly involving the frontal and temporal regions [1,2,3]. More specifically, several studies have shown abnormalities in WM integrity in patients with schizophrenia (SZPs) compared to healthy controls (HCs), particularly in the fronto–temporo-limbic pathways (e.g., bilateral superior longitudinal and inferior fronto-occipital fasciculi), interhemispheric connections (e.g., the *Corpus callosum* (CC) and the fornix), and cortico-cerebellar tracts (e.g., bilateral cortico-spinal tracts) [4,5,6], confirmed by several meta-analyses [7,8]. Furthermore, these WM alterations are associated with both positive (e.g., hallucinations, delusions) and negative symptoms (e.g., social withdrawal, avolition, anhedonia, alogia, blunted affect) and disorganized speech and behavior in SZPs [9,10,11]. Additionally, antipsychotics may have differential impacts on WM [12,13], with no or limited effects on negative symptoms and cognition [14,15,16]. Life expectancy in SZPs is reduced by 15–20 years because of high cardiovascular mortality related to a sedentary lifestyle, poor dietary habits, elevated substance use disorders and the cardiometabolic side effects of antipsychotics [17,18,19,20]. Adjuvant non-pharmacological interventions are needed for this population, and lifestyle interventions such as physical activity (PA) have emerged as an effective and safe strategy [21]. Indeed, studies have demonstrated that PA significantly improves cardiovascular capacities in SZPs by reducing weight, insulin resistance, lipidic dysregulation, type II diabetes and metabolic syndrome, leading to decreased mortality [22,23,24,25].

To our knowledge, few studies have investigated the effect of PA on WM integrity and connectivity. Svatkova et al. [26] demonstrated that PA significantly increased the integrity of tracts involved in motor functions, such as the body of the corpus callosum (BCC) and the left corticospinal tract (CST), suggesting an increase in structural brain connectivity. These authors also highlighted a reduction in positive symptom severity following a PA program that was significantly correlated with WM integrity improvement, based on fractional anisotropy (FA). Additionally, in their cross-sectional study, Maurus et al. [27] found trends toward positive associations between aerobic fitness and WM volumes in the left parahippocampus and several cerebellar regions in SZPs.

In the present study, we assessed changes in WM integrity in SZPs and HCs following an adapted PA program delivered remotely by web (e-APA). We also investigated associations among changes in WM integrity, cardiorespiratory fitness and symptom severity after e-APA.

## 2. Materials and Methods

### 2.1. Experimental Design and Participants

The present study came from longitudinal, interventional, open-label trial [28], registered at ClinicalTrial.gov (accessed on 16 August 2017, NCT03261817). All data were collected at baseline and after the 16-week intervention (post-intervention endpoint). SZPs were diagnosed based on the Diagnostic and Statistical Manual of Mental Disorders (DSM-IV-TR, 4th edition, text revision) and the structured Mini International Neuropsychiatric Interview (MINI, French version 5.0.0). Eighteen eligible SZPs were recruited based on inclusion and exclusion criteria (Table 1), and 13 eligible HCs were then matched to SZPs based on PA level, using the Ricci and Gagnon (RG) self-questionnaire scores (very active: >35; active: 18–35; inactive: <18) and on age and gender.

All participants were free of auditory deficits, neurological disorders and cerebral abnormalities and gave their informed written consent in accordance with the Declaration of Helsinki. Patients were stabilized outpatients with no change in their antipsychotic treatment over the last two months and during the entirety of the protocol. The study protocol was approved by the French health authority, the Agence Nationale de Sécurité du Médicament et des produits de santé (ANSM) on 10 July 2016 (ID-RCB number 2016-A00930-51). Also, the study protocol was approved by the local ethics committee (Comité de Protection des Personnes; CPP Nord-Ouest IV, France; CPP 16/39) on 13 January 2017 in compliance with French regulations.

### 2.2. e-APA Intervention

All patients participated in the e-APA program via real-time videoconferencing using a secure web-based platform called VisioMoov, managed by Mooven^®^, as explained in our previous studies [28,29]. Each patient completed two 60-min sessions per week over a period of 16 weeks, totaling 32 sessions. Throughout the sessions, patients interacted with a qualified APA professional coach via videoconference.

The e-APA program was developed according to the guidelines from the World Health Organization (WHO) and the American College of Sports Medicine (ACSM), based on the recommendations from expert committees [21,30,31,32]. It included three types of exercises: predominantly aerobic (e.g., running/walking on the spot, step exercises, jumping jacks, dancing), resistance (e.g., planks, push-ups, burpees, squats) and balance exercises (e.g., standing crunch with under-the-leg clap, single-leg cross-body punches). Each session lasted 40 min, with additional warm-up and cool-down periods before and after each session, respectively (5–10 min each). The exercises were performed at a moderate-to-vigorous intensity, aiming to reach 60–75% of the maximal heart rate, which was determined by each patient’s baseline maximal exercise test and monitored through heart rate recording during the workouts.

Each participant’s exercise was personalized and adapted on their baseline fitness level, sporting history, existing pain, ongoing treatment and personal preferences or motivation. A motivational interview and verbal feedback were conducted at both the beginning and the end of each session, allowing for the adjustment of exercise intensity according to the participants’ fitness levels and any potential pain.

### 2.3. Cardiorespiratory Fitness

Cardiorespiratory fitness was assessed by measuring maximal oxygen uptake (VO_2_max, mL/min/kg) during a cardiac stress test under medical supervision in both sessions (baseline and post-intervention). Measurement was taken using Ergoline er900^®^ equipment (ergoline moving to health, Bitz, Germany) and participants had to pedal as long as possible while the load increased by 20 watts every 2 min. O_2_ flow rate was recorded before the beginning of the test (resting values), at each level (relative values), until the end of the test (maximal values) and for the following 3 min of passive recovery (recovery values).

### 2.4. Clinical Symptoms

In SZPs, symptom severity was assessed using the Positive and Negative Syndrome Scale (PANSS) [33], which includes a total score and three subscores regarding positive symptoms, negative symptoms and general psychopathology. Each subscale consists of several items rated on a 7-point Likert scale. Negative symptoms were also evaluated using the Self-Evaluation of Negative Symptoms (SNS) [34] and the Brief Negative Syndrome Scale (BNSS) [35]. The SNS is a self-report measure that includes 20 items covering five domains such as social withdrawal, diminished emotional expression, anhedonia, alogia and avolition. Participants rate the severity of each symptom on a scale from 0 to 2. The BNSS is a hetero-evaluation consisting of 13 items that assess five domains: anhedonia, asociality, avolition, blunted affect and alogia. Each item is rated on a scale from 0 to 6. For all these scales, higher scores indicate a greater severity of symptoms.

### 2.5. Neuroimaging

#### 2.5.1. Data Acquisition

Diffusion tensor imaging data were acquired using a 3-Tesla scanner (Intera Achieva 3T Quasar Dual, Philips Medical System, Andover, MA, USA). The two-session (baseline and post-intervention) imaging protocol included diffusion-weighted images (DWI) from 48 diffusion gradient directions, with two images without diffusion weighting and two images with either a postero-anterior or antero-posterior phase-encoding direction (EPI-SE diffusion sequence; factor b = 1000 s/mm^2^; TR/TE = 9937/62 ms; 90° FA; 128 × 128 × 60 matrix; slice thickness = 2 mm; 256 mm FOV; 2-mm isotropic voxel size; transverse slice orientation; SENSE factor = 2).

#### 2.5.2. Data Processing

Diffusion data preprocessing involved using the standard pipeline from the FMRIB Software Library (FSL, Oxford, UK, http://www.fmrib.ox.ac.uk/fsl; v6.0.5, accessed on 16 August 2017) [36] and FMRIB’s Diffusion Toolbox (FDT). This process included: (1) correcting susceptibility-induced distortions using TOTUP [37]; (2) brain extraction with the brain extraction tool (BET); (3) correcting eddy current–induced distortions and motions using EDDY [38,39,40]; and (4) fitting the diffusion tensor model to preprocessed data using DTIFIT to generate diffusion maps of FA and radial diffusivity (RD in mm^2^/s) for each participant.

For data post-processing and to investigate the influence of e-APA on WM integrity, we conducted an analysis using tract-based spatial statistics (TBSS) from FSL [41]. The steps of TBSS included: (1) erosion of each participant’s FA maps; (2) nonlinear and linear registrations to align all FA maps into a common space (FMRIB58_FA and MNI152); (3) creation of a mean FA image from the previously transformed maps, which was then skeletonized to generate a skeleton of WM pathways (representing the central voxels of the pathways common to all participants), using a threshold value of FA 0.3 to exclude gray matter and cerebrospinal fluid-containing voxels; (4) creation of individual FA skeletons by projecting participants’ FA data onto the population-specific mean FA skeleton; and (5) mapping of RD values onto the population-specific mean FA skeleton using the projection vectors from each participant’s FA-to-skeleton transformation. These steps were necessary to conduct voxel-wise statistical analyses.

### 2.6. Statistical Analyses

All statistical analyses, except for the TBSS analyses, were performed using JMP v13.0 Software (SAS Institute, Inc., Cary, NC, USA) or JASP v0.18.3. The significance level was set at *p* < 0.05.

#### 2.6.1. TBSS Analyses

To assess diffusion differences in skeleton voxels between groups (SZPs vs. HCs), we conducted statistical tests using the general linear model from FSL implemented with the randomize tool for nonparametric permutation inference [42]. A correction by threshold-free cluster enhancement (TFCE) [43] was applied and was, in conjunction, either corrected for multiple comparisons with family-wise error (*p*_FWE_ < 0.05, TFCE) or not corrected (*p* < 0.05, TFCE) using 5000 permutations. The significant regions (*p* < 0.05) were identified using the Johns Hopkins University—International Consortium of Brain Mapping tract and label atlases [44]. Both groups were compared at baseline using a two-sample unpaired *t*-test adjusted for RG score, age and gender. Relative variation (RV) maps were generated to assess longitudinal changes. RV was taken as the difference between skeletonized diffusion maps in post-intervention and skeletonized diffusion maps at baseline, divided by skeletonized diffusion maps at baseline. This calculation was performed using the FSLmaths tool. A two-sample unpaired *t*-test, adjusted for RG score, age and gender, was used on the RV maps to assess for a diagnostic effect (SZPs vs. HCs) on WM integrity changes. Post-hoc analyses (paired *t*-tests) were carried out to assess these changes in each group (SZPs and HCs) between baseline and post-intervention.

To illustrate TBSS results, mean diffusion values (FA and RD) for each participant were extracted in the WM skeleton pre- and post-intervention for all significant regions and exported to JMP v13.0 or JASP v0.18.3 software. In the context of physiological interpretation, better integrity was characterized by higher FA (axonal development) and lower RD (better myelination) [45,46,47].

#### 2.6.2. Correlation Analyses

Within-group correlation analyses (Spearman) between RV of clinical/VO_2_max and diffusion data were conducted to evaluate the effect of e-APA over time on the relationships between symptom severity/cardiorespiratory fitness and WM integrity. Correlations were considered statistically significant at *p* < 0.05 after Bonferroni correction for multiple comparisons (*p* = 0.0071 (0.05/7) for all clinical data (total, positive, negative and general-PANSS, SNS, BNSS) and VO_2_max). Mean diffusion values were extracted only in significant contrast maps from previous TBSS analyses. 

## 3. Results

### 3.1. Per Protocol Population Characteristics

Among the 31 participants, following the e-APA program, four (2 SZPs and 2 HCs) did not complete the study and two (1 SZP and 1 HC) were excluded because of motion artefacts on imaging. Two HCs did not have a DWI acquisition because of a scanner problem. Therefore, the per protocol population consisted of 15 SZPs and 8 HCs. These two groups differed significantly in RG scores at baseline (Table 2). Post-intervention VO_2_max values were missing for two SZPs.

### 3.2. Diffusion Tensor Imaging

#### 3.2.1. Baseline

**Figure 1:**
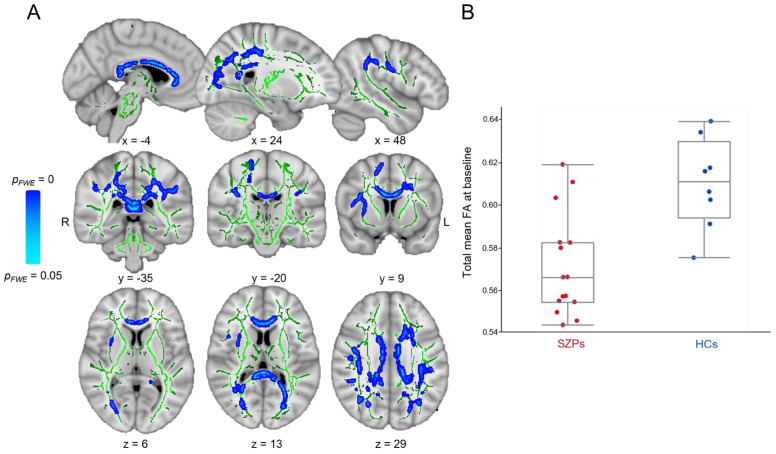
White matter (WM) integrity difference at baseline between patients with schizophrenia (SZPs) and healthy controls (HCs). (**A**) Fractional anisotropy (FA) was significantly increased in HCs compared to compared to SZPs following a tract-based spatial statistics analysis (in blue/light blue; *p* < 0.05, TFCE and adjusted for Ricci and Gagnon scores). Statistical analysis was conducted within the population-specific WM skeleton (in green), and Montreal Neurological Institute coordinates are displayed. (**B**) Total mean FA across all clusters in both groups. L: left; R: right.

TBSS analysis revealed that compared to SZPs, HCs had significantly increased FA (*p*_FWE_ < 0.05, TFCE) in many WM fasciculi or regions (Figure 1A and Table 3) with a higher total mean FA value (HCs: median [quartile 1; quartile 3] = 0.61 [0.63; 0.59]; SZPs: 0.57 [0.58; 0.55]; Figure 1B). In addition, RD was significantly reduced in HCs compared to SZPs (*p*_FWE_ < 0.05, TFCE; total mean RD value in HCs: 0.49 10^−3^ [0.52 10^−3^; 0.47 10^−3^]; SZPs: 0.54 10^−3^ [0.55 10^−3^; 0.52 10^−3^]). These RD decreases were highlighted in the same clusters as FA and in additional regions (Table 4).

#### 3.2.2. Longitudinal

**Figure 2:**
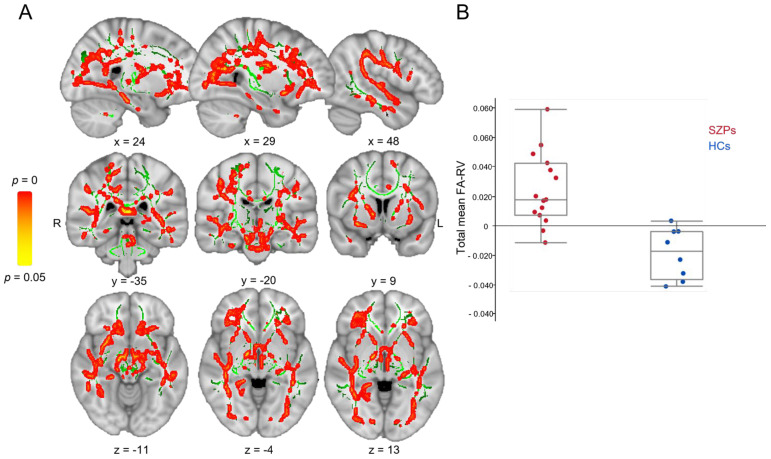
Effect of adapted physical activity (e-APA) on white matter (WM) integrity changes in patients with schizophrenia (SZPs) compared to healthy controls (HCs). (**A**) SZPs had significantly increased fractional anisotropy relative variation (FA-RV = changes between baseline and post-intervention) compared to HCs following e-APA program with a tract-based spatial statistics analysis (in red–yellow; *p* < 0.05, TFCE and adjusted for Ricci and Gagnon score). Statistical analysis was conducted within the population-specific WM skeleton (in green), and Montreal Neurological Institute coordinates are displayed. (**B**) Total mean FA-RV across all clusters in both groups. L: left; R: right.

SZPs had clusters with significantly increased FA-RV (*p* < 0.05, TFCE; Figure 2A) in comparison to HCs (total mean FA-RV value in SZPs: 0.018 [0.043; 0.0077]; HCs: −0.017 [−0.0035; −0.036]; Figure 2B and Table 3). SZPs also had decreased RD-RV compared to HCs (*p* < 0.05, TFCE; total mean RD-RV value in SZPs: −0.014 [0.0050; −0.081]; HCs: 0.072 [0.091; 0.047]; see Table 4).

**Figure 3:**
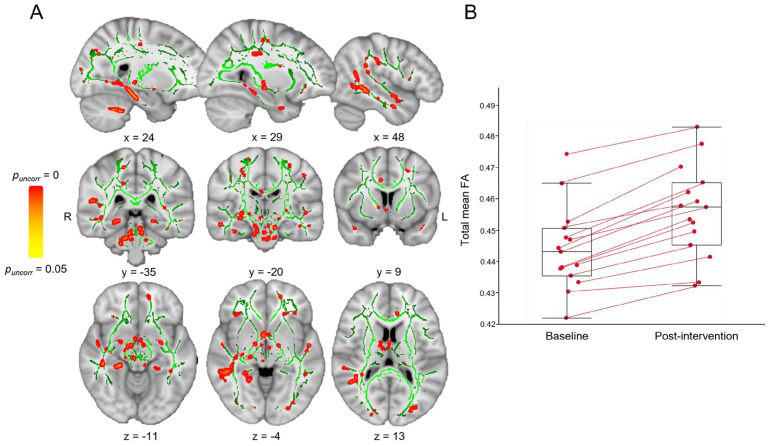
Effect of adapted physical activity (e-APA) on white matter (WM) integrity in patients with schizophrenia (SZPs) before and after program. (**A**) SZPs had significantly increased fractional anisotropy (FA) in post-intervention compared to FA value at baseline with a tract-based spatial statistics analysis (in red–yellow; *p* < 0.05, TFCE). Statistical analysis was conducted within the population-specific WM skeleton (in green), and Montreal Neurological Institute coordinates are displayed. (**B**) Total mean FA across all clusters in SZPs in baseline and post-intervention. L: left; R: right.

Regarding pre- and post-intervention analyses, we demonstrated an FA increase after program only in SZPs and not found in HCs (*p* < 0.05, TFCE; Figure 3A and Table 3) with a total mean FA value higher in post-intervention than in baseline (baseline: 0.44 [0.43; 0.45], post-intervention: 0.46 [0.45; 0.47]; Figure 3B). SZPs also displayed a decreased RD after program e-APA, not found in HCs (*p* < 0.05, TFCE; baseline: 0.54 10^−3^ [0.52 10^−3^; 0.54 10^−3^], post-intervention: 0.52 10^−3^ [0.50 10^−3^; 0.52 10^−3^]; Table 4).

### 3.3. Relationships between White Matter Integrity and Cardiorespiratory Fitness/Clinical Data

We found a negative correlation between BNSS RV and r-inferior longitudinal fasciculus (ILF) FA-RV (*p* = 0.023, rho = −0.58). However, this correlation was not significant after Bonferroni correction (*p* < 0.0071). No other significant relationship was observed.

## 4. Discussion

Our findings highlight a positive effect of e-APA on WM integrity in SPZs, suggesting improved brain connectivity.

At baseline, SZPs had lower FA and higher RD compared to HCs, consistent with existing literature indicating WM integrity abnormalities in schizophrenia in a diffuse cortico-subcortical network [1,2,3,7]. Moreover, following the e-APA program, only SZPs had a significant FA increase and/or RD decrease, suggesting an axon myelination process in various WM fasciculi and notably in those altered at baseline. These fasciculi included the CST, BCC and middle cerebellar peduncle (CP), which are involved in motor functions that are engaged during PA practice. Indeed, the CST links the motor cortex, encompassing the supplementary motor area and the primary motor and premotor cortices, which are responsible for movement planning, coordination and voluntary movement control. It extends from these regions to the spinal cord, traversing through the internal capsule [48]. The regions of the motor cortex are interconnected in a homologous manner between the two hemispheres through the BCC, which plays a role in motor integration. The middle CP is involved in fine motor coordination, movement error correction and maintaining balance [49]. Thus, the CST, BCC and middle CP together may play a role in controlling motricity and mobilization during e-APA sessions, improving WM integrity in SZPs.

Previous studies have demonstrated a positive effect of physical exercise on these brain structures [50,51,52]. For example, Ikuta and Loprinzi [50] found a positive association in the CST between FA and PA, assessed using the International Physical Activity Questionnaire and a negative association with RD, suggesting that PA improved WM integrity in this fasciculus. The results of the present study indicated greater WM integrity improvement in the superior longitudinal fasciculus (SLF), which is involved in regulating motor behavior through its part I [53,54]. In addition, WM integrity improvement after the e-APA program in SZPs included other fiber bundles usually altered in schizophrenia such as the uncinate, inferior fronto-occipital fasciculi, ILF, SLF (especially its segment III, known as the arcuate fasciculus), the splenium of the CC and the cingulum bundle, which are involved in language and/or cognitive processes known to be altered in SZPs [55,56,57,58,59].

Taken together, these results suggest that brain plasticity mechanisms in SZPs are preserved and that e-APA may contribute to improved brain connectivity, in accordance with Svatkova et al. [26]. They reported a positive effect of PA regardless of diagnosis; however, we identified this effect only in patients, likely because of the smaller number of HCs. Several mechanisms may underlie the WM integrity improvement identified here, including decreased inflammation, increased blood flow and increases in growth factors such as brain-derived neurotrophic factor, which in turn can boost neurogenesis and gliogenesis [51,60]. Additionally, these different mechanisms could promote proliferation and differentiation of oligodendrocyte precursor cells, ultimately leading to enhanced bundle myelination [61].

We also highlighted a negative relationship between BNSS score and the right ILF FA over time, suggesting that WM integrity improvement in the right ILF might induce a reduction in negative symptom severity in SZPs who practice e-APA. This implication is supported by the fact that the ILF plays a role in socio-emotional behavior, which is related to negative symptoms [62].

Ultimately, our findings may have potential clinical implications for the management of schizophrenia. The observed negative relationship between BNSS score and the right ILF FA suggests that e-APA intervention aimed at improving WM integrity may contribute to an improvement of negative symptoms in SZPs. Indeed, negative symptoms, such as blunted affect, avolition, anhedonia and social withdrawal, are related to socio-emotional behavior that involve the ILF. Increasing the WM integrity in this brain region might lead to improve the negative symptoms. However, future research should investigate the specific mechanisms underlying these relationships and explore the potential of APA as an adjunctive treatment for the negative symptoms of schizophrenia.

Although the current study offers promising findings, it has limitations. Given the small sample size, it is important to consider this study exploratory. Nevertheless, this study is necessary as it addresses the nearly non-existent literature on the effects of APA on WM integrity. Among the limitations is a relatively small sample size, so that most of the results were uncorrected by FWE. Thus, further researches with a larger number of participants will be needed to validate and generalize our findings. It should be noted that the small size of the sample of HCs primarily resulted from logistical constraints, particularly associated with the employment status of the participants which prevented their participation for a long period (5 months) required by the protocol. Additionally, the COVID-19 pandemic (the period when HCs could be recruited), the participant withdrawals and the loss of imaging data also contributed to the reduction in sample size. Also, the lack of significant improvement in WM integrity in HCs following the e-APA program may be attributed to several factors. First, the relatively small size of the sample of HCs in the study may have limited the statistical power to detect significant effects. While previous studies have indicated a positive impact of PA on WM integrity in HCs [63,64], the extent of this enhancement may be lower compared to SZPs due to inherent differences. Specifically, HCs generally have higher baseline levels of physical activity than patients, which may result in a ceiling effect, making it more difficult to achieve further improvements. Additionally, the concept of dose response in PA interventions [65,66] suggests that HCs may need more intense and prolonged exercise to achieve measurable changes in WM integrity. In our present study, exercises were tailored to the patients’ physiological fitness level (“adapted”), which may not have been intense enough to induce changes in HCs. However, although the lack of significant findings in HCs warrants further investigation, it is plausible that the effects of APA on WM integrity differ between SZPs and HCs. Additionally, we did not test axial diffusivity (AD), also known as parallel diffusivity, which is defined as the diffusion of water along the direction of the principal diffusion. This decision was made due to its lack of sensitivity in regions with branching and crossing WM fibers as found in the intra-hemispheric tracts included in the present study [45]. A NODDI (Neurite Orientation Dispersion and Density Imaging) approach could have provided a more detailed assessment of WM integrity, particularly in regions with complex fiber arrangements. However, we did not initially acquire multi-shell DTI sequences in our study, given the prolonged acquisition time required for our patient population. We also did not test mean diffusivity (MD) that represents the average diffusion of water molecules along the fiber because of its less specific sensitivity to myelin alterations than RD (perpendicular diffusion). Ultimately, we did not compare the e-APA effects with another intervention in SZPs that did not allow us to conclude a more specific effect of e-APA on the WM integrity in patients. Therefore, future studies incorporating a standard control condition could elucidate the specific effects of e-APA in schizophrenia.

## 5. Conclusions

These findings contribute to the growing body of literature on how PA improve brain connectivity in SZPs. Our results indicate that e-APA improved WM integrity abnormalities observed in SZPs, suggesting preserved brain plasticity mechanisms in this population. Remote delivery by web of an APA program offers a convenient and accessible approach that could enhance treatment adherence in SZPs, making it a promising therapeutic strategy. Nevertheless, further investigations into the specific mechanisms underlying these WM integrity changes, as well as their functional and/or clinical implications, are warranted to advance understanding of how PA is beneficial in schizophrenia.

## Figures and Tables

**Table 1:** Inclusion and exclusion criteria for participants from our previous study [28].

**Inclusion Criteria** For all participants (SZPs and HCs): Age range: 18 to 60 years old Signed informed consent Ability to receive intervention: possession of a computer, internet access and a webcam Medical welfare affiliation *For SZPs:* Meeting DSM-IV-TR criteria for schizophrenia or schizoaffective disorders Stable psychotropic medication for at least 2 months prior to inclusion Guardian or trustee consent for protected majors
**Exclusion criteria** For all participants (SZPs and HCs): Under 18 or over 60 years old Participation in another biomedical research protocol during the present study Pregnancy MRI contraindications Progressive neurological diseases Physical restrictions on physical activity (moderate to severe heart failure, unstable coronary disease, severe valvular disease, recent pulmonary embolism or untreated deep venous thrombosis, uncontrolled hypertension, pulmonary arterial hypertension treated or not) Neuromuscular disorders, severe sensory and/or motor neuropathy Rheumatic and articular disorders; orthopedic/rheumatologic problems or bone lesions at risk of fracture that contraindicate physical activity History of stroke or myocardial infarction within 6 months prior to the selection visit *For HCs:* Lifetime diagnosis of schizophrenia or schizoaffective disorder according to the DSM-IV-TR criteria

DSM-IV-TR: Diagnosis and Statistical Manual of Mental Disorders, 4th edition, text revision; HCs: healthy controls; MRI: magnetic resonance imaging; SZPs: patients with schizophrenia.

**Table 2:** Per protocol population characteristics at baseline.

Median (Quartile 1; Quartile 3) [Min–Max]	SZPs	HCs	*p*-Value
Number of participants, *n*	15	8	-
Gender (males, *n* (%))	11 (73.3)	5 (62.5)	0.59
Handedness (right/left/both, *n* (%))	13/1/1 (86.7/6.7/6.7)	6/1/1 (75/12.5/12.5)	0.78
Age (years)	40.5 (32.8; 47.3) [25.8–55.1]	39 (35.8; 46.6) [34.0–50.6]	0.72
Education level (years)	12 (11; 16) [7–20]	15 (12; 16.8) [11–20]	0.16
RG’s class (active/inactive, *n* (%))	9/6 (60/40)	7/1 (87.5/12.5)	0.17
RG score	20 (13; 25) [9–30]	27.5 (22.5; 31.3) [13–33]	0.045
VO_2_max (mL/min/kg)	22.5 (19.1; 30) [16.5–36.8]	32.1 (24.7; 47.7) [16.4–49.7]	0.076
Age of disease onset (years)	20 (19; 23) [17–30]	-	-
Illness duration (years)	17 (11; 24) [4–35]	-	-
Chlorpromazine equivalent (mg/day)	325 (140; 400) [50–800]	-	-
Total PANSS	59 (48; 71) [44–96]	-	-
Positive PANSS	13 (8; 15) [7–23]	-	-
Negative PANSS	17 (12; 23) [10–32]	-	-
General PANSS	31 (24; 35) [19–44]	-	-
SNS	14 (10; 15) [3–23]	-	-
BNSS	22 (10; 41) [5–53]	-	-

BNSS: Brief Negative Syndrome Scale; HCs: healthy controls; m: mean; *n*: number of participants; PANSS: Positive and Negative Syndrome Scale; RG: Ricci and Gagnon; SNS: Self-Evaluation of Negative Symptoms; SZPs: patients with schizophrenia. Significance level at *p* < 0.05. To examine differences between groups, the Mann–Whitney U test was used for noncategorical variables and the chi-square test for categorical variables.

**Table 3:** Significant white matter (WM) fasciculi and regions grouped into clusters from fractional anisotropy (FA) TBSS analyses.

WM Fasciculi or Regions from JHU-ICBM Tracts and Labels Atlases Number of Voxels (>100) (Proportion in %)	FA—Baseline HCs > SZPs *p*_FWE_ < 0.05, TFCE	FA-RV—Longitudinal SZPs > HCs *p* < 0.05, TFCE		SZPs Post-Intervention > Baseline *p* < 0.05, TFCE
	JHU-tracts			
L-anterior thalamic radiation	353 (0.22)	871 (0.89)		140 (0.61)
R-anterior thalamic radiation	213 (0.12)	1263 (1.83)		254 (1.00)
L-corticospinal tract	102 (0.19)	427 (0.59)		168 (0.73)
R-corticospinal tract	411 (0.88)	709 (0.68)		462 (1.21)
L-cingulum gyrus	440 (0.36)	286 (0.30)		-
R-cingulum gyrus	185 (0.19)	174 (0.058)		-
R-cingulum hippocampus	-	339 (0.32)		198 (1.29)
Forceps major	1091 (2.48)	1286 (1.49)		-
Forceps minor	845 (4.06)	525 (1.21)		-
L-inferior fronto-occipital fasciculus	-	794 (1.42)		-
R-inferior fronto-occipital fasciculus	575 (1.10)	1837 (2.61)		130 (0.82)
L-inferior longitudinal fasciculus	147 (0.17)	763 (1.20)		193 (1.06)
R-inferior longitudinal fasciculus	109 (0.52)	1385 (1.20)		322 (1.82)
L-superior longitudinal fasciculus	892 (2.25)	1095 (0.90)		-
R-superior longitudinal fasciculus	1450 (3.32)	2039 (1.94)		241 (1.68)
R-superior longitudinal fasciculus- part temporal	-	110 (0.63)		150 (0.83)
L-uncinate fasciculus	-	331 (0.54)		-
R-uncinate fasciculus	-	332 (0.45)		-
	JHU-labels			
Genu of the corpus callosum	790 (8.00)	-		-
Body of the corpus callosum	1260 (12.78)	132 (0.71)		-
Splenium of the corpus callosum	1125 (11.34)	672 (3.65)		-
L-corona radiata	502 (5.05)	250 (1.35)		-
R-corona radiata	143 (1.44)	516 (2.79)		-
L-sagittal stratum	-	127 (0.69)		-
R-sagittal stratum	-	209 (1.13)		-
L-external capsule	-	276 (1.06)		-
R-external capsule	-	399 (1.01)		-
R-anterior internal capsule	-	231 (1.25)		-
R-posterior internal capsule	-	142 (0.77)		-
L-retrolenticular part of internal capsule	-	223 (1.21)		-
Middle cerebellar peduncle	-	174 (0.94)		399 (10.07)
L-cerebellar peduncle	-	123 (0.66)		-
R-cerebellar peduncle	-	169 (0.92)		-

HCs: healthy controls; JHU-ICBM: Johns Hopkins University–International Consortium of Brain Mapping; L: left; R: right; RV: relative variation; SZPs: patients with schizophrenia.

**Table 4:** Significant white matter (WM) fasciculi and regions grouped into clusters from radial diffusivity (RD) TBSS analyses.

WM Fasciculi or Regions from JHU-ICBM Tracts and Labels Atlases Number of Voxels (>100) (Proportion in %)	RD—Baseline HCs > SZPs *p*_FWE_ < 0.05, TFCE	RD-RV—Longitudinal SZPs > HCs *p* < 0.05, TFCE		SZPs Post-Intervention > Baseline *p* < 0.05, TFCE
	JHU-tracts			
L-anterior thalamic radiation	1096 (0.49)	741 (1.26)		629 (1.44)
R-anterior thalamic radiation	1555 (1.03)	953 (1.38)		554 (1.49)
L-corticospinal tract	334 (0.15)	423 (0.47)		855 (1.85)
R-corticospinal tract	1488 (0.86)	662 (0.77)		670 (1.30)
L-cingulum gyrus	1034 (0.40)	-		-
R-cingulum gyrus	559 (0.14)	-		-
R-cingulum hippocampus	137 (0.033)	351 (0.50)		231 (0.81)
Forceps major	2395 (1.38)	1035 (1.92)		221 (0.65)
Forceps minor	3009 (3.53)	-		-
L-inferior fronto-occipital fasciculus	993 (0.76)	340 (0.91)		-
R-inferior fronto-occipital fasciculus	2875 (1.93)	1050 (2.41)		185 (0.81)
L-inferior longitudinal fasciculus	469 (0.31)	418 (1.00)		337 (1.50)
R-inferior longitudinal fasciculus	2043 (1.25)	1071 (1.87)		504 (1.55)
L-superior longitudinal fasciculus	3420 (1.82)	310 (0.38)		266 (0.56)
R-superior longitudinal fasciculus	4161 (2.10)	1191 (1.36)		196 (0.66)
R-superior longitudinal fasciculus—part temporal	122 (0.63)	226 (0.62)		120 (0.48)
L-uncinate fasciculus	276 (0.28)	-		-
R-uncinate fasciculus	531 (0.37)	239 (0.48)		-
	JHU-labels			
Genu of the corpus callosum	1176 (3.17)	-		-
Body of the corpus callosum	1899 (5.11)	-		-
Splenium of the corpus callosum	1491 (4.00)	355 (3.04)		-
L-corona radiata	1504 (4.04)	-		-
R-corona radiata	1568 (4.21)	237 (1.95)		-
R-sagittal stratum	184 (0.49)	121 (0.99)		-
L-external capsule	300 (0.79)	-		-
R-external capsule	618 (1.17)	438 (2.18)		-
R-anterior internal capsule	305 (0.80)	116 (0.95)		-
R-posterior internal capsule	330 (0.88)	117 (0.96)		-
L-retrolenticular part of internal capsule	-	115 (0.94)		-
R-retrolenticular part of internal capsule	199 (0.53)	-		-
Middle cerebellar peduncle	-	604 (4.95)		1019 (15.68)
R-cerebellar peduncle	224 (0.59)	155 (1.27)		-

HCs: healthy controls; JHU: Johns Hopkins University–International Consortium of Brain Mapping; L: left; R: right; RV: relative variation; SZPs: patients with schizophrenia.

## Data Availability

The data presented in this study are available on request from the corresponding author due to legal reasons.
